# Psychological Impact of Treatment-Induced Erectile Dysfunction on Masculinity: A Study of a Group of Black Elderly Men Undergoing Prostate Cancer Treatment at a Tertiary Hospital in Limpopo Province, South Africa

**DOI:** 10.3390/ijerph23010110

**Published:** 2026-01-16

**Authors:** Shai Nkoana, Tholene Sodi, Antonio Lentoor, Mokoena Maepa, Kgadi Thobejane

**Affiliations:** 1 Department of Psychology, University of Limpopo, Sovenga 0727, South Africa; 2 DSI/NRF-UL SARChl (Mental Health), University of Limpopo, Polokwane 0727, South Africa; 3 Department of Clinical Psychology, Sefako Makgatho Health Sciences University, Pretoria 0204, South Africa

**Keywords:** prostate cancer, Black men, erectile dysfunction, hegemonic masculinity, health related quality of life

## Abstract

With emerging improvement in screening and treatment, most patients with prostate cancer (PCa) live for a long period beyond their diagnosis. Erectile dysfunction (ED) and bowel and urinary incontinence have been shown to be the most bothersome side-effects of most PCa treatment options for patients. With increasing long-term survival, most PCa patients face the prospect of experiencing symptoms, side-effects of available treatment options, and diminished quality of life. The objective of the study was to explore the impact of treatment-induced ED on masculinity among Black South African PCa patients. Twenty (20) prostate cancer patients, selected through purposive sampling and receiving some form of treatment at Pietersburg tertiary Hospital, with ages ranging from 67 to 85 years (mean age = 76 years; SD = 5.3), participated in the study. In-depth, individual semi-structured interviews were used to collect data and analyzed through Interpretative Phenomenological Analysis (IPA). The findings indicate that ED threatens or adversely impacts the participants’ perceptions of their sense of masculinity leading to diminished quality of life. The results demonstrate that loss of masculinity brought about by PCa treatment-induced ED is experienced both physically as well as psychologically. The results highlight a need for collaboration between medical and psychological professionals in the management of PCa patients. This is crucial for improving the overall health related quality of life for patients.

## 1. Introduction

The global burden of prostate cancer PCa among the population of men is recognized as an increasing global public health concern [1,2,3]. Black men have the highest disproportional prevalence of PCa than any other racial group across the globe [4]. The reasons for the disproportionate burden of PCa among men of African ancestry remain unclear [5]. In South Africa, PCa is the most common cancer in men across all population groups with Black men having the highest incidences than any other racial group [6,7,8,9]. Equally concerning is the fact that Black men frequently present with more advanced and aggressive stage of the disease at diagnosis [6,7]. Some of the reasons for Black men presenting late at PCa diagnosis and with more advanced stage of the disease include limited access to healthcare [10], lack of awareness of screening and early detection programs [7,11], and poor or lack of knowledge of the disease among Black communities in South Africa [1,10,12]. Additionally, Black men are also less likely to participate in clinical research, making it difficult to understand the true burden associated with PCa within this population group [13].

At present, with improvement in screening practices and advancement in treatment options, most PCa patients now live for a decade or longer following diagnosis in both high-income countries as well as low-income and middle-income countries [14,15,16]. As more men diagnosed with PCa live for a long period beyond their diagnosis, attention is increasingly shifting to the psychological issues associated with the disease [15,17]. A focus on patients’ quality of life, including health-related quality of life, is fast gaining momentum within oncological research and clinical practice [18]. PCa survivorship and managing side effects of available treatment options has never been more important [19].

There are a variety of treatment options available for PCa patients. These different treatment options are usually based on the stage of the PCa at diagnosis [15]. According to [15], the primary goal of PCa treatment is to cure, prolong survival, or aid in palliative care. Unfortunately, each PCa treatment option has its own side-effects. ED is one of the major complications of most PCa treatments and is of importance to a significant proportion of patients and their partners [20,21,22]. Lehto et al. [17] put it succinctly thus: “All active prostate cancer treatments result in sexual difficulties” (P. 865). ED is the inability to achieve and maintain an erection sufficient enough for satisfactory sexual activity, either occasionally or consistently [15]. Due to treatment-induced ED, PCa patients’ masculinity (i.e., a sense of being a man) may be adversely affected [21]. In some societies, including in Africa, ideas about masculinity and what it means to be a ‘man’ are intimately tied to certain cultural expectations, norms, and practices [23]. According to [20], sexuality is not an isolated phenomenon but rather an integral part of a man’s identity and lifestyle. Enactment of gender scripts, that is, ways of thinking, feeling, and acting are based on culturally prescribed norms of masculinity. PCa treatment induced ED may disrupt the identity of men who define masculinity through (hetero) sexual penetrative intercourse and performance.

The theory of hegemonic masculinity contents that masculinity is a culturally constructed ideal of what it means to be a man [21]. It proposes that masculinity is not biologically determined but a socially constructed concept that emphasizes the interplay between men’s identity, ideals, interactions, power, and patriarchy [23]. Framed within the theory of hegemonic masculinity, the aim of this study was to investigate how Black South African PCa survivors understand the impact of their PCa disease, including treatment side-effects, on their masculinity and self-identity.

## 2. Methods

### 2.1. Study Design

The study was part of a bigger project investigating the lived experiences of a group of Black South African PCa patients. The study utilized a hermeneutic phenomenological study design. Hermeneutic studies are concerned with the scientific investigation of human experiences as it is lived [24]. According to Smith [25], human beings create meanings in the different experiences that shape their lives.

### 2.2. Sampling

The study utilized a purposive sampling method to select twenty (*n* = 20) Black South African men who were receiving some form of PCa treatment at a tertiary hospital in Limpopo Province, South Africa. The treatment options the patients were receiving included surgery, hormone therapy, radiotherapy, and chemotherapy. Both practical as well as theoretical considerations formed the basis of the sample size as well as selection procedure. According to Smith and Osborn [26], a distinctive feature of phenomenological inquiry is its commitment to detailed account of participants’ narrative account of their experiences, and this can only be achieved with a small sample.

### 2.3. Individual Interviews

In-depth, individual semi-structured interviews were conducted with 20 Black South African PCa survivors who were receiving some form of treatment at Pietersburg Provincial Hospital. The hospital is a tertiary institution where PCa patients from the different parts of the Province of Limpopo receive oncology treatment. Inclusion criteria included the following: Black South African citizen, PCa diagnosis for at least five years before the interview, and able to communicate in one of the most commonly spoken languages in the Province of Limpopo, i.e., English, Sepedi, Xitsonga, and Tshivenda. Exclusion criteria included the following: individuals who fulfill the inclusion criteria but not wishing to take part, who had mental health problems that may exacerbate any adverse feelings that may arise during the interviews, and those who did not speak any of the identified local languages. Each individual interview was audio recorded (after permission was obtained from each participant) and transcribed verbatim (with review by an independent bilingual expert) from the local languages of each participant into English for the broader scientific community.

Semi-structured interviews allow space for rich, detailed, first-person accounts of human experience, which the researcher may inquire in detail with prompt probing questions. An interview guide, with a set of questions, was used to collect data. Each individual interview took approximately one hour to complete. The interview guide included some of the following statements: tell me about your experiences with prostate cancer disease and share with me how you cope with the disease, including treatment side-effects.

### 2.4. Data Analysis

IPA was used to analyze the data obtained in the study. The main strength of IPA is its ability to give full account of each participant’s personal experience [25]. The primary aim of IPA is to explore, in detail, how each participant makes sense of their experiences. The method is compatible with hermeneutic phenomenology. The method (IPA) was deemed appropriate because the study was interested in understanding the experiences of living with PCa-induced side-effects by the participants. Each case-by-case analysis was conducted by the primary researcher who is experienced in the art of IPA.

### 2.5. Ethics

Prior to the commencement of the study, permission was obtained from the following institutional bodies: the University of Limpopo Turfloop Research Ethics Committee (TREC/26/2015) and Limpopo Provincial Department of Health Ethics Committee (Ref:4/2/2) as well as a gatekeeper permission from Pietersburg Provincial Hospital (Ref:2/8/2). All the participants in the study signed informed consent forms.

### 2.6. Trustworthiness of the Study

Credibility, transferability, dependability, and conformity (to ensure trustworthiness) were observed in the study. All the five research team members (experienced clinical psychologists) cross-checked the data analysis and involved themselves in a reflective engagement of a dialog with the participants’ narratives and meanings to ensure trustworthiness of the study.

## 3. Results

All the participants in the study were diagnosed with PCa for at least five years (>5 years) before the commencement of the study. All were men between 67 and 85 years of age (*n* = 20; mean age =76; SD = 5.3) and receiving some form of treatment. The majority had primary school education (*n* = 15), retired (*n* = 13), and receiving government social security grant (*n* = 7). All, except one, had no family history of prostate cancer. All participants had poor knowledge of PCa and had never participated in any screening for the risk of prostate cancer prior to their formal diagnosis. In their majority (*n* = 16), the participants reported ED and differing loss of their masculinity (*n* = 14).

The table below (Table 1) highlights some of the participants’ narratives about the impact of PCa treatment-induced ED on their masculinity. All the participants (whose narrative quotes are reflected in Table 1) indicated during their respective individual interviews that they did not experience ED prior to their PCa diagnosis and/or treatment commencement. Subsequently, a discussion of the findings is offered.

## 4. Discussion

The aim of this study was to explore the impact of PCa-induced ED on masculinity among Black South African patients. All the participants (Black PCa patients) were receiving some form of treatment (unspecified) for their condition at the time of the study. These included surgery, hormone therapy, radiotherapy, and chemotherapy. The results (as reflected by the varied representative quotes of the participants) show that the majority of PCa patients experienced ED. This negatively impacted their sense of masculinity. ED was talked about by the participants in the study in terms of emasculation (i.e., the inability to achieve an erection and thereby penetrative sex).

According to Brüggemann [27], hegemonic masculinity emphasizes the pursuit of toughness and self-reliance as the cornerstones of what it means to be a man. This pursuit is achieved through certain gender role expectations that are socialized early in life through which men internalize certain attitudes and behaviors associated with masculinity [21]. Consequently, treatment-induced ED may threaten PCa patients’ overall sense of masculinity. According to Chambers et al. [14], hegemonic masculinity (i.e., a man’s identity—a sense of them being a man) may be linked to how men perceive themselves and respond to PCa diagnosis and treatment, including their psychological adjustment. Sexual virility in men—the ability to complete conjugal duty through (hetero)sexual penetrative intercourse, is frequently considered an important aspect in traditional hegemonic masculine practices and ideals [27]. According to Walther et al. [22], PCa survivors may experience what is termed “double jeopardy” (i.e., they are first affected by the failure of erectile functioning and then by accompanying psychological distress of losing their masculinity.

The participants in the study narrated stories of unhappiness and a threat to their masculinity as a result of their inability to attain an erection as a result of PCa-induced ED. This is consistent with what has been found elsewhere in other studies. For example, Bekele et al. [28] found in their study that PCa patients linked their penile erection (as well as sexual performance) to their masculine identity. In another study, Neenanz and Chatz [29] found that PCa and its treatment can have a negative effect on patients’ body image, sexual function, and (masculine) identity. In their majority, the participants in the study narrated a loss of their masculinity (‘I am not a man’) as a result of their ED. Through the participants’ narrative stories, the participants demonstrated that sexual function is a central theme to perceptions of masculinity. The shame (‘I can’t talk about it’) evidenced in some participants’ narratives demonstrate that this threat to their masculinity created a vicious cycle and a conflation of the diminished erectile function and loss of manhood. This subsequently led to their questioning of their self-worth (‘I am nothing’). This suggests that participants in the study viewed their self-worth in terms of their sexual potency and the ability to achieve an erection.

Men in the study valued sexual potency or virility as a script indicative of their masculinity. As a result, the men in the study construed failure to have an erection as unmasculine consequently leading to feelings of shame and humiliation. This is akin to a feeling of victimization experience that threatens men socially constructed masculine identity. The findings in this study are consistent with previous research identifying that ED does negatively impact perceptions of masculinity [14,21,22,23,27,28,29,30]. Additionally, most of the participants expressed a sense of “double jeopardy” (i.e., physical failure of the erectile functioning rendering penetrative sexual intercourse impossible as well as the associated psychological distress due to the loss of masculinity) that has been documented in other previous studies elsewhere [20,21,27,28,31].

As demonstrated in the study, the preservation of erectile function is an important concern for PCa patients. As the number of patients surviving PCa continues to increase due to improvements in diagnosis and treatment options, the number of men concerned with ED post-treatment will also continue to increase. Due to the connection between PCa treatment side-effects and ED, there is a need to explore strategies for managing ED [32]. There is lack of research studies on treatment options for ED following PCa treatment [32,33,34]. These studies [32,33,34] suggest that a substantial proportion of men do not access ED help to improve sexual function after PCa treatment. Managing both physical as well as psychological implication of ED is paramount [33,34]. Whilst ED following PCa treatment remains a concern within the scientific community, some promises regarding the management of ED are emerging. These include penile implants [35], penile rehabilitation, testosterone replacement therapy, vacuum constriction devices, intracavemosal injections, oral PDE5 inhibitors [32,34,35], as well as psychosexual therapy [35].

## 5. Conclusions

Based on the results of the study, it is evident that for most participants, masculinity is a critical factor in their experience of PCa treatment-induced ED. Hegemonic masculinity appears to frame how the men in the study interpret what is happening to them. For the men in the study, masculinity suffered tremendous harm as a result PCa treatment-induced ED leading to diminished quality of life. Impotence and the inability to achieve an erection due to treatment induced ED can be received as a sexual failure and a threat to a man’s hegemonic masculinity. Our study suggests that in addition to the physical loss of sexual function through ED, many men undergoing PCa treatment experience adverse psychological distress, leading to decreased health-related quality of life. Treatment-induced ED poses serious threats to men’s scripts for hegemonic masculinity.

How masculinity is impacted by treatment-induced ED still remains unclear. There is, therefore, a need for empirical research to establish or identify factors that may interact with masculinity as a mediator of psychological outcomes for patients experiencing PCa-induced ED. Equally important, it is best to measure the baseline erectile function of PCa patients before treatment decisions are made. For example, patients may be asked about erectile function during pre-treatment discussions with their medical physicians.

Given the role of masculinity as a critical factor in men’s perceptions of ED after PCa treatment, consideration should be given to holistic management interventions that are responsive to hegemonic masculinity. Patients diagnosed with PCa should receive psychoeducation about ED risks of all treatment options available to them and the potential impact of ED on their post-treatment quality of life. This calls for a collaboration between medical and psychological professionals for optimal management of PCa patients.

## 6. Limitations of the Study

Participants were elderly PCa patients, and the results may not be generalizable to younger population groups among Black South African men. Despite this, the results in this study may further contribute to the body of literature on the impact of different PCa treatment options on masculinity as well as the overall quality of life of patients, which is currently lacking within the South African context.

## Figures and Tables

**Table 1:** Quotes highlighting damage/loss of masculinity narrated.

Participants	Representative Quotes
B	“I always knew what to do with my wife in bed…. When this illness hit you, you lose that control. Now I am just a parcel. I’m nothing”.
F	“…Another problem is in bed. I have not slept with my wife (have sex) for a very long time. I am a man…even if I am old. This is a big worry”
J	“I have not discussed this with my wife…but every time I try, nothing happens. You reach a stage where you wonder whether you are still a man. This is a big problem. I mean …. I want to satisfy my wife and sleep with her. I married her as my wife. Married people should sleep together. That is all.”
M	“You know…since my operation, this is a problem. You see…you are no longer a real man when you can’t have it up. You think about it …and nothing. All energy is gone. It is a tough thing. You just sleep there. I hear this thing castrate you. I don’t know myself.”
P	“I can’t talk to anyone about it. You just stay with it. I mean…. what will they think of me? I tried to drink some things but…but no nothing. I am not a man anymore”
K	“I do not have dignity anymore. I am not a man like any man… I mean, if you even wear nappies and your urine just come. What life is that?

## Data Availability

The data used in the study are available on request from the corresponding author. The data are not publicly available due to the ethical nature of the study, which did not include data sharing.
